# Drug-induced metabolic syndrome hasn’t associations with 5-HT receptor genes polymorphisms in patients with schizophrenia

**DOI:** 10.1192/j.eurpsy.2021.1027

**Published:** 2021-08-13

**Authors:** D. Paderina, I. Pozhidaev, A. Bocharova, E. Kornetova, A. Boiko

**Affiliations:** 1 Laboratory Of Molecular Genetics And Biochemistry, Mental Health Research Institute, Tomsk National Research Medical Center of the Russian Academy of Sciences, Tomsk, Russian Federation; 2 Laboratory Of Evolutionary Genetics, Research Institute of Medical Genetics, Tomsk National Research Medical Center of the Russian Academy of Sciences, Tomsk, Russian Federation; 3 Mental Health Research Institute, Tomsk National Research Medical Center of the Russian Academy of Sciences, Tomsk, Russian Federation; 4 Laboratory Of Molecular Genetics And Biochemistry, Mental Health Research Institute,Tomsk National Research Medical Center of the Russian Academy of Sciences, Tomsk, Russian Federation

**Keywords:** Serotonin receptors, Metabolic syndrome, schizophrénia, Genes

## Abstract

**Introduction:**

Metabolic disturbances are common in patients maintained on neuroleptics. These abnormalities significantly increase the physical comorbidity and mortality rates due to cardiovascular disease. We hypothesized that 5-HT receptor genes polymorphisms have associations with drug-induced metabolic syndrome development in schizophrenic patients.

**Objectives:**

To investigate the role of polymorphic variants of serotonin receptors genes in the development of antipsychotic-induced metabolic syndrome.

**Methods:**

467 patients with schizophrenia receiving long-term antipsychotic therapy were investigated. The mean age was 40.0±11.6 years. The standard phenol-chloroform method for DNA isolation was used. Genotyping was carried out on eight SNP’s of genes HTR1A, HTR2A, HTR3A and HTR2C with the MassARRAY^®^ Analyzer 4 (Agena Bioscience™) using the set SEQUENOM Consumables iPLEX Gold 96 on the base The Core Facility “Medical Genomics”, Tomsk NRMC.

**Results:**

The prevalence of metabolic syndrome was 26.1%. In the study sample, there were significantly more women with metabolic syndrome (56.6%) than men (43.4%) (p=0.002). The majority of patients with metabolic disturbances were aged >40 years (62.3%), versus 40.9% in the group without metabolic disorders (p<0.001). The duration of the disease was statistically significantly higher in the group of patients with metabolic syndrome (p=0.003). We did not find statistically significant associations of polymorphic variants of the studied genes with the development of the drug-induced metabolic syndrome.

**Conclusions:**

Our results do not demonstrate any significant association between allelic variants of serotonin receptor genes and metabolic syndrome in patients with schizophrenia. Conflict of interest. The authors declare no conflict of interest. Supported by Grant of RSF 19-75-10012.

